# Short communications: an ear-attached accelerometer detects effects of regrouping on lying, rumination, and activity times in calves

**DOI:** 10.1007/s11259-023-10151-9

**Published:** 2023-07-01

**Authors:** N. Ramezani Gardaloud, C. Guse, L. Lidauer, A. Steininger, F. Kickinger, M. Öhlschuster, W. Auer, M. Iwersen, M. Drillich, D. Klein-Jöbstl

**Affiliations:** 1 Clinical Unit for Herd Health Management in Ruminants, Department for Farm Animals and Veterinary Public Health, University Clinic for Ruminants, University of Veterinary Medicine, 1210 Vienna, Austria; 2 Smartbow GmbH / Zoetis LLC, Jutogasse 3, 4675 Weibern, Austria

**Keywords:** Accelerometer, Regrouping, Calf, Lying time, Rumination time

## Abstract

The objective of this study was to use a sensor-based accelerometer (ACC) to identify changes in lying, rumination, and activity times in weaned calves during the moving and regrouping process. Overall, 270 healthy Holstein calves (from approximately 16 regrouping events) at the age of approximately 4 months were enrolled and equipped with an ear-attached ACC (SMARTBOW, Smartbow GmbH/ Zoetis LLC). Sensor data were recorded for 5 d before (d -5) until 4 d after moving and regrouping (d 4). The day of regrouping was defined as d 0. Acceleration data were continuously processed by specific algorithms (developed by SMARTBOW) for lying, rumination, and activity. Lying, rumination, and activity times were averaged from d -5 to d -3 to generate a baseline value for each parameter. Parameters on d 0 to d 4 after regrouping were compared to this baseline. All parameters showed significant changes compared with the baseline at d 0. Significant decreases in rumination and inactive times were seen up to d 2. Lying time was significantly lower until d 3. The study results indicate that the ACC can be used to monitor the disruptive effects of regrouping on lying and rumination behaviors. Further research is necessary to elucidate how these changes have an impact on health, performance, and welfare and to evaluate how to reduce these potentially negative effects.

**Supplementary Information:**

The online version contains supplementary material available at 10.1007/s11259-023-10151-9.

## Introduction

Regrouping and moving is a common management practice during calf rearing. It is defined as moving and mixing (un)familiar animals from one pen to another, based on, e.g., age or sex. Mixing and regrouping of animals can influence their behavior, which could have negative consequences on animal performance and well-being (Grant and Albright, 2001, Von Keyserlingk et al., 2008). Regrouped animals re-establish social relationships using physical and nonphysical communication (Arave and Albright, 1976), leading to social and individual distress (Von Keyserlingk et al., 2008). In adult cows, regrouping has a negative effect, e.g., on milk production, feeding time, and behavior (Arave and Albright, 1976, Hasegawa et al., 1997, Von Keyserlingk et al., 2008). This social instability, especially if done too often and unpredictably might also increase the risk of disease, although, to the best of our knowledge, there is no study proofing this hypothesis (Proudfoot and Habing, 2015). Negative effects can last for hours (Lecorps et al., 2020) or up to days (Grant and Albright, 2001, Von Keyserlingk et al., 2008). Previous studies mainly focused on the influence of regrouping on milk production and behavior in cows, i.e., feeding, rumination, lying, social behavior, and anhedonia (Grant and Albright, 2001, Schirmann et al., 2011, Lecorps et al., 2020). In calves, only few studies are available, mainly focusing on behavior, e.g., sniffing and aggression, general activity (Veissier et al., 2001), and lying times (Lindner et al., 2021). Behavioral changes have been suggested as a potentially useful measure of animal welfare (Mattiello et al., 2019). Hence, evaluating how regrouping affects the calves’ behavior might be of importance with regard to animal welfare and future performance. In recent years, new technologies like accelerometers (ACC) have been used as a reliable and affordable technology to monitor behavioral changes. These technologies to detect behavioral changes even in larger groups of calves may overcome group size limitation of visual monitoring (Wurtz et al., 2019).

The aim of the study was to use an ear-tag attached ACC to evaluate lying, rumination, and activity times in calves after moving and regrouping. To our knowledge this is the first time that an ACC system was used to monitor these parameters in calves after regrouping. We hypothesized that the ACC is able to detect effects of moving and regrouping, including the return to a baseline value.

## Materials and methods

### Animals, experimental design and housing

The study procedures were approved by the Slovakian Regional Veterinary Food Administration and noted by the institutional ethics and animal welfare committee of the University of Veterinary Medicine, Vienna (ETK-11/09/2017). This study was part of a larger project conducted on a commercial dairy farm in Slovakia over a period of 11 mo. The study farm housed approximately 2,700 Holstein dairy cows and young stock. For the first 8 wk. of life, all calves were housed in individual hutches (123 cm **×** 154 cm, installed with a distance from the bottom of the hutches to the barn floor of 25 cm), equipped with approximately 15 kg of dry and hygienic wheat or oat straw as bedding material. Calves had visual and direct contact via a grit to their neighbor calves. Calves were fed from buckets with a rubber teat twice per day (at 6:00 and 14:00) with milk and milk replacer at an amount of at least 4L/meal. After milk feeding calves had access to fresh water via the milk feeding bucket. All calves had ad libitum access to starter concentrate. After weaning calves were housed in groups of five animals of the same age in pens (3m **×** 4.8m = 2.8 m^2^/calf) in a barn with natural ventilation. Four weeks later the calves were grouped, within the same barn in group pens of 10 animals (6m **×** 4.8m = 2.8 m^2^/calf). Calves were grouped randomly from different groups. At the age of approximately 4 months, calves were moved and regrouped in pens of approximately 30 animals (7.7×13.5 m = 3.5 m^2^/calf) in another barn on the same farm site. Every week, a total of 60–70 calves of 6–7 groups, were randomly selected by farm staff and moved into two large groups of approximately 30 calves by truck (in the study barn). Due to the random selection of animals, each calf was grouped with familiar and unfamiliar calves at an unknown proportion. After removing older calves to another heifer rearing farm, each pen was cleaned and afterwards a novel new group of approximately 30 animals entered the study barn. Consequently, calves entering the study barn were exposed to changes in the physical and social environment. Regrouping took place between 10:00 a.m. and 12:00 a.m. and took overall (loading on the hanger, transport, and unload from the hanger) about 1 h. All calves, during their stay in the groups of 10 calves, were equipped with an ear-attached ACC approximately 2–3 weeks before moving to the study barn. In all group pens (from 5 to 30 animals), animals were kept on deep straw bedding. Calves had free access to water and feed, fed freshly twice daily (at 07:00 and 16:00). Calves received a total mixed ration for ad libitum intake consisting of 33% corn silage, 17% grass silage, 16% concentrates, 9% wet distillers grain with soluble, 8.5% beet pulp, 6% corn cob mix, 5% straw, 4% soy extraction meal, and 1.5% minerals. All calves were clinically examined, i.e., assessment of fever, nasal and eye discharge, coughing and lung abnormality, other abnormalities e.g., diarrhea, by one veterinarian (first author N.R.G) at the day of moving to and regrouping in the study barn and only healthy animals were selected for the present study. Overall, the data of 458 healthy calves at the age of 126.4 (± 14.5) days were used for analysis, while the sample size for each separate day was different (see suppl. table1).

### Accelerometer

All calves in the small group barn (n = 10) were equipped with a 10 Hz ear-attached accelerometer (SMARTBOW, Smartbow GmbH / Zoetis LLC, Weibern, Austria) with a size of 52 × 36 × 17 mm and a weight of 34 g. Receiver devices (Smartbow Wallpoints) sent data in real time to a local server (Smartbow FarmServer). The continuously recorded acceleration data were further processed on a local server and the classified data were available on the farm computer or mobile devices, or both. The algorithms used in this study were originally developed for cows (Reiter et al. 2018) and evaluated for detecting selected behaviors in calves (with sensitivity 94.4%, specificity 94.3%, precision 95.8%, and accuracy 94.3%, and Cohen’s kappa of 0.88 for lying vs. standing and for defined activities; the overall accuracy was 70.8% and kappa 0.58 (Roland et al. 2018b). Based on these algorithms, classified data for lying, rumination, and activity levels (i.e., active, inactive, high active) for each calf were continuously recorded (min/h) in the SMARTBOW software. Regarding activity levels an animal could either be active, inactive, or high active, and the sum of these three parameters was always 60 min/h. The algorithms and classification of lying, rumination, and activity level are intellectual property of the company.

### Statistical analyses

All statistical analyses were performed using the Python packages pandas; SciPy and NumPy (Python 3.7.9, pandas 1.3.5, SciPy 1.7.3 (Virtanen et al., 2020), and NumPy 1.21.5 (Harris et al., 2020)), and R environment version 4.0.2 including the ggplot 2 package 3.3.2 (Wickham and Chang, 2016). ACC data labelled as valid by SMARTBOW (i.e., at least data for at least 40 out of 60 min were available) were selected and summarized per calf. Before analysis, the average times (min/h) for lying, rumination, inactive, active, and high active were calculated for each calf for 5 d before (d -5) until 4 d after moving and regrouping (d 4), separately. The day of regrouping was defined as d 0. For this, we calculated the mean by just summing up all values for each day per calf (24h) and then divided the sum by the number of values (24), resulting in min/h. For each measure a mean ± standard deviation (SD) was calculated. To describe a baseline value, we used the three consecutive days before d 0 (d -5 to d -3) with the highest number of available datapoints (270 calves) in small group pens of 10 calves. Durations of lying, rumination, and activity times per day between the baseline and the days 0 to 4 were compared using a linear mixed effects models with the day as fixed and the animals as random effect. The level of significance was set at *P <* 0.05.

## Results

**Fig. 1:**
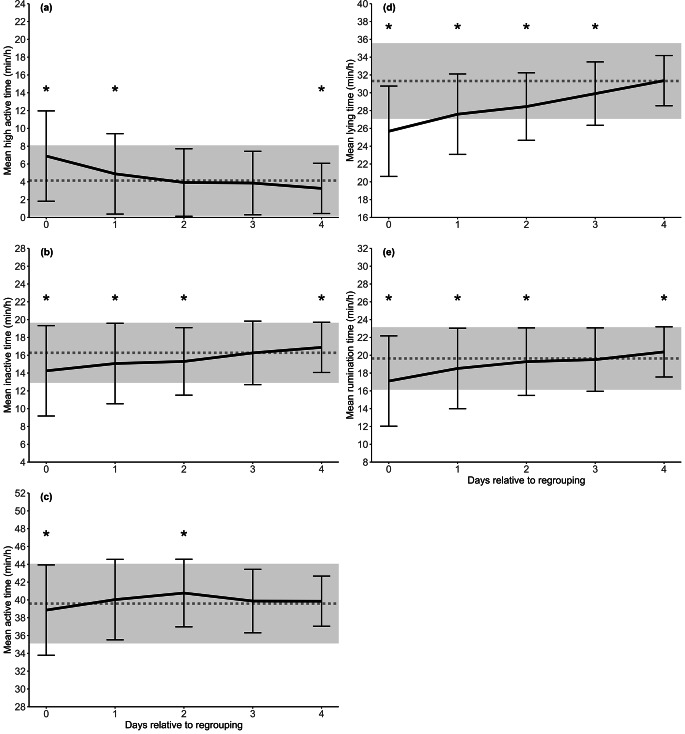
Means (± standard deviation, SD) for (**a**) high active, (**b**) inactive, (**c**) active, (**d**) lying, and (**e**) rumination time (min/h) compared to a baseline (dotted line, with grey area presenting SD). Lying, rumination, and activity times were averaged from d -5 to d -3 to generate a baseline value for each parameter. Presented are day 0, the day of regrouping, and the following 4 days. * Significant differences based on linear mixed effect models

Lying, rumination, and activity times of the baseline, day 0 and for the days 1 to 4 after regrouping are presented in Fig. 1. Generally, all measured parameters differed significantly from the baseline on the day of regrouping (d 0). Mean lying (25.7 ± 5.5 min/h), rumination (17.1 ± 3.7 min/h), active (38.9 ± 4.9 min/h), and inactive (14.3 ± 3.6 min/h) times were shorter on d 0 compared to the baseline. At the same time calves had longer high active times (6.9 ± 5.1 min/h vs. 4.1 ± 4.0 min/h for baseline) at the day of regrouping. The reduction in rumination and inactive times remained lower for the following 2 d compared to the baseline (Fig. 1d and 1b). Reduction in lying times remained significantly lower on d 1, d 2 and d 3 (Fig. 1e). Calves that were moved and regrouped to a new pen showed a gradual increase in lying, inactive, and rumination times on the days following d 0, and inactive and rumination times were even significantly longer on d 4 compared to the baseline. Activity patterns (i.e., active, inactive, and high active) differed significantly during the following days after regrouping.

## Discussion and conclusion

Previous studies showed that the used ACC is able to monitor lying (sensitivity (SE) = 94%, specificity (SP) = 94% ) (Roland et al., 2018b) and drinking behavior (SE 83%, SP 97%) in calves (Roland et al., 2018a). Although the algorithms used in the current study were not validated for calves, it has been shown that ACC system has the potential, e.g., to detect early indicators for newborn calf diarrhea (SE 68%, SP 62%) (Goharshahi et al., 2021). In this study we were able to show, that moving and regrouping had a significant effect on the calves’ lying, rumination, and activity times classified by the ACC. As expected, regrouping led to shorter inactive and lying times, which lasted for 4 days. Shorter lying times could be explained by increasing times moving around the barns, exploring the surroundings, interacting with new herd mates or developing social stability (Veissier et al., 2001). Some authors described an effect of regrouping day on lying times in heifers (Mazer et al., 2020), while others reported few to no change in dairy cows (Von Keyserlingk et al., 2008, Schirmann et al., 2011). Longer high active times at the day of moving and regrouping is probably related to chasing, merging and moving of animals by truck. Herskin et al. (2004) reported that rumination, which is considered as an indicator of stress, showed a decrease in stressful situation. This might also be true for the stressful situation of regrouping and moving in our study. Schirmann et al. (2011) reported a decrease in time spent ruminating following regrouping in dairy cows. Interestingly, lying and rumination times were increased significantly on d 4 compared to the baseline. This may be a sign of adaptability to changes, overcoming negative effects like moving and regrouping. Furthermore, improvement in housing, in this case by more space allowance per calf may lead to a fast adaptation. In summary, calves adapted and values returned to baseline levels within a few days. The fast return to baseline is in accordance with other studies, where animals also adapted within approximately 3 days (Von Keyserlingk et al., 2008). In another study these changes lasted up to 15 days (Hasegawa et al., 1997). Although in our study the calves experienced introduction to a new group of animals and replacing into another barn, including transportation at the same time, the examined parameters went back to baseline levels within a few days. A possible explanation is that the calves in the current study already experienced moving and regrouping before. Hence, one would assume that this can lead to a certain degree of familiarization to such a situation (Foris et al., 2021). In this study we were able to monitor a large number of animals and groups by using an ACC, adressing the limitation in other studies (Foris et al., 2021). Nevertheless, this current study has its limitations. We did not test social networks within regrouping process. Further work is needed to perform continuous video observation to achieve a detailed picture about social interactions e.g., aggression and dominance behavior that may have an influence on lying, rumination, and activity and familiarization in entire group.

In conclusion, we were able to support the hypothesis that moving and regrouping to a new pen has a significant effect on the calves’ activity. Detecting changes in group housed calves on the days following regrouping is particularly challenging, due to difficulties of observing individual animals. Hence the used ACC are increasing the ability to detect and follow up these changes.

### Electronic supplementary material

Below is the link to the electronic supplementary material.

Supplementary Material 1

## Data Availability

All data generated during the study are available from the corresponding author on reasonable request upon approval by the commercial partner.

## References

[CR1] Arave CW, Albright JL (1976). Social rank and physiological traits of dairy cows as influenced by changing group membership. J Dairy Sci.

[CR2] Foris B, Haas HG, Langbein J, Melzer N (2021). Familiarity influences social networks in dairy cows after regrouping. J Dairy Sci.

[CR3] Goharshahi M, Azizzadeh M, Lidauer L, Steininger A, Kickinger F, Öhlschuster M, Auer W, Klein-Jöbstl D, Drillich M, Iwersen M (2021). Monitoring selected behaviors of calves by use of an ear-attached accelerometer for detecting early indicators of diarrhea. J Dairy Sci.

[CR4] Grant RJ, Albright JL (2001). Effect of animal grouping on feeding behavior and intake of dairy cattle. J Dairy Sci.

[CR5] Harris CR, Millman KJ, Van Der Walt SJ, Gommers R, Virtanen P, Cournapeau D, Wieser E, Taylor J, Berg S, Smith NJ (2020). Array programming with NumPy. Nature.

[CR6] Hasegawa N, Nishiwaki A, Sugawara K, Ito I (1997). The effects of social exchange between two groups of lactating primiparous heifers on milk production, dominance order, behavior and adrenocortical response. Appl Anim Behav.

[CR7] Herskin MS, Munksgaard L, Ladewig J (2004). Effects of acute stressors on nociception, adrenocortical responses and behavior of dairy cows. Physiol Behav.

[CR8] Lecorps B, Weary DM, von Keyserlingk MA (2020). Regrouping induces anhedonia-like responses in dairy heifers. JDS Commun.

[CR9] Lindner EE, Gingerich KN, Miller-Cushon EK (2021). Effects of early social contact on dairy calf response to initial social grouping and regrouping. J Dairy Sci.

[CR10] Mattiello S, Battini M, De Rosa G, Napolitano F, Dwyer C (2019). How can we assess positive welfare in ruminants? Animals.

[CR11] Mazer KA, Knickerbocker PL, Kutina KL, Huzzey JM (2020). Changes in behavior and fecal cortisol metabolites when dairy cattle are regrouped in pairs versus individually after calving. J Dairy Sci.

[CR12] Proudfoot K, Habing G (2015). Social stress as a cause of diseases in farm animals: current knowledge and future directions. Vet J.

[CR13] Reiter S, Sattlecker G, Lidauer L, Kickinger F, Öhlschuster M, Auer W, Schweinzer V, Klein-Jöbstl D, Drillich M, Iwersen M (2018). Evaluation of an ear-tag-based accelerometer for monitoring rumination in dairy cows. J Dairy Sci.

[CR14] Roland L, Lidauer L, Sattlecker G, Kickinger F, Auer W, Sturm V, Efrosinin D, Drillich M, Iwersen M (2018). Monitoring drinking behavior in bucket-fed dairy calves using an ear-attached tri-axial accelerometer: a pilot study. Comput Electron Agric.

[CR15] Roland L, Schweinzer V, Kanz P, Sattlecker G, Kickinger F, Lidauer L, Berger A, Auer W, Mayer J (2018). Evaluation of a triaxial accelerometer for monitoring selected behaviors in dairy calves. J Dairy Sci.

[CR16] Schirmann K, Chapinal N, Weary DM, Heuwieser W (2011) and M. A. G. Von Keyserlingk. Short-term effects of regrouping on behavior of prepartum dairy cows. J. Dairy Sci 94(5):2312–2319 10.3168/jds.2010-3639 21524520

[CR17] Veissier I, Boissy A, dePassillé AM, Rushen J, Van Reenen CG, Roussel S, Andanson S, Pradel P (2001). Calves’ responses to repeated social regrouping and relocation. J Anim Sci.

[CR18] Virtanen P, Gommers R, Oliphant TE, Haberland M, Reddy T, Cournapeau D, Burovski E, Peterson P, Weckesser W, Bright J (2020). SciPy 1.0: fundamental algorithms for scientific computing in Python. Nat Methods.

[CR19] Von Keyserlingk MAG, Olenick D, Weary DM (2008). Acute behavioral effects of regrouping dairy cows. J Dairy Sci.

[CR20] Wickham H, Chang W (2016) Create Elegant Data Visualisations Using the Grammar of Graphics Version 2(1):1–189

[CR21] Wurtz K, Camerlink I, D’Eath RB, Fernández AP, Norton T, Steibel J, Siegford J (2019). Recording behaviour of indoor-housed farm animals automatically using machine vision technology: a systematic review. PLoS ONE.

